# miR-431 secreted by human vestibular schwannomas increases the mammalian inner ear’s vulnerability to noise trauma

**DOI:** 10.3389/fneur.2023.1268359

**Published:** 2023-10-09

**Authors:** Takeshi Fujita, Richard Seist, Shyan-Yuan Kao, Vitor Soares, Lorena Panano, Radhika S. Khetani, Lukas D. Landegger, Shelley Batts, Konstantina M. Stankovic

**Affiliations:** ^1^Department of Otolaryngology – Head and Neck Surgery, Massachusetts Eye and Ear and Harvard Medical School, Boston, MA, United States; ^2^Department of Biostatistics, Harvard T.H. Chan School of Public Health, Boston, MA, United States; ^3^Department of Otolaryngology-Head and Neck Surgery, Stanford University School of Medicine, Stanford, CA, United States; ^4^Department of Neurosurgery, Stanford University School of Medicine, Stanford, CA, United States; ^5^Wu Tsai Neuroscience Institute, Stanford University, Stanford, CA, United States

**Keywords:** vestibular schwannoma, extracellular vesicles, hearing loss, micro RNA, noise trauma

## Abstract

**Introduction:**

Vestibular schwannoma (VS) is an intracranial tumor that arises on the vestibular branch of cranial nerve VIII and typically presents with sensorineural hearing loss (SNHL). The mechanisms of this SNHL are postulated to involve alterations in the inner ear’s microenvironment mediated by the genetic cargo of VS-secreted extracellular vesicles (EVs). We aimed to identify the EV cargo associated with poor hearing and determine whether its delivery caused hearing loss and cochlear damage in a mouse model *in vivo.*

**Methods:**

VS tissue was collected from routinely resected tumors of patients with good (VS-GH) or poor (VS-PH) pre-surgical hearing measured via pure-tone average and word recognition scores. Next-generation sequencing was performed on RNA isolated from cultured primary human VS cells and EVs from VS-conditioned media, stratified by patients’ hearing ability. microRNA expression levels were compared between VS-PH and VS-GH samples to identify differentially expressed candidates for packaging into a synthetic adeno-associated viral vector (Anc80L65). Viral vectors containing candidate microRNA were infused to the semicircular canals of mice to evaluate the effects on hearing, including after noise exposure.

**Results:**

Differentially expressed microRNAs included hsa-miR-431-5p (enriched in VS-PH) and hsa-miR-192-5p (enriched in VS-GH). Newborn mice receiving intracochlear injection of viral vectors over-expressing hsa-miR-431-GFP, hsa-miR-192-GFP, or GFP only (control) had similar hearing 6 weeks post-injection. However, after acoustic trauma, the miR-431 group displayed significantly worse hearing, and greater loss of synaptic ribbons per inner hair cell in the acoustically traumatized cochlear region than the control group.

**Conclusion:**

Our results suggest that miR-431 contributes to VS-associated hearing loss following cochlear stress. Further investigation is needed to determine whether miR-431 is a potential therapeutic target for SNHL.

## 1. Introduction

Vestibular schwannoma (VS), the fourth most common intracranial tumor, typically arises from the vestibular portion of cranial nerve VIII and presents with sensorineural hearing loss in 95% of affected patients (1). Although VS is not malignant, it is associated with substantial morbidity because it can also cause tinnitus, dizziness, facial paralysis, and other cranial neuropathies (1). VS’s unchecked expansion into the cerebellopontine angle and associated brainstem compression may become life-threatening (2).

The etiology of VS-induced SNHL was long believed to be compression of the adjacent auditory nerve by the VS tumor, but multiple studies including diverse cohorts have failed to find a correlation between sporadic VS tumor size and the degree of a patient’s SNHL (3–8), and have observed that progressive SNHL may occur without VS growth (4). Post-mortem examinations of untreated VS patients have revealed cochlear damage ipsilateral to the tumor, including loss of sensory inner hair cells (IHC), outer hair cells (OHC), and innervating spiral ganglion neurons (SGNs) in 75, 88, and 85% of patients, respectively (6, 7), suggesting that cochlear damage contributes to VS-induced hearing loss. Intriguingly, patients with VS also often experience declines in hearing ability and cochlear innervation contralateral to the tumor where auditory nerve compression cannot occur (9, 10).

There is emerging evidence of differential gene expression and protein production in VS tumors from patients with preserved auditory function [good hearing (GH)] and those with severe-to-profound SNHL [poor hearing (PH)] (11). For example, mouse cochlear explants exposed to human VS-PH tumor secretions displayed hair cell loss and neuronal fiber disorganization that were positively associated with the VS donors’ degree of SNHL, while explants exposed to VS-GH tumor secretions displayed no damage or solely neuronal fiber disorganization (11). Similarly, a transcriptomic analysis of human VS tumors and secretions identified differential expression and proteolytic activity of matrixmetalloprotease 14 (MMP-14) in tumors associated with SNHL (12). The application of MMP-14 to mouse cochlear explants led to damage to SGNs, including their ribbon synapses with hair cells. Additionally, proteins related to the NACHT, LRR, and PYD domains-containing protein 3 (NLRP3) inflammasome (i.e., NLRP3 and interleukin [IL]-1β) are preferentially present in the tumor specimens of VS-PH patients (13), while fibroblast growth factor 2 (FGF-2) is secreted at higher levels by VSs from patients with GH than PH (14). Thus, the contributions of VS to SNHL are currently presumed to be multi-factorial and to involve tumor-induced changes to the inner ear’s microenvironment, cellular viability, and gene expression.

A potential mechanism facilitating VSs’ negative impact to hearing is the secretion of exosomes, such as extracellular vesicles (EVs), which contain ototoxic cargo. EVs are lipid-membrane vesicles measuring ~30–200 nm and are released into the extracellular space by most cell types, including normal and neoplastic cells (15). They are involved in a diverse range of biological processes but primarily function to facilitate cell–cell communication by transferring their cargo of DNA, RNA, and proteins from one cell to another, locally or systemically (16). The RNA within EVs can be functional and the delivered micro RNA (miRNA) can regulate protein translation in target cells, including in the inner ear during development, response to acoustic trauma and inflammation, and aging (17, 18). Importantly, EVs have been implicated in immune signaling, tumorigenesis, and metastasis in cancer, whereby the uptake of tumor-derived EVs can alter a cell’s phenotype to cancerous by prompting or inhibiting gene transcription (16, 19–23).

Using a dual culture system, we have previously reported that EVs are produced by VSs and can be internalized by cochlear cells *in vitro*, with varying effects depending on the hearing status of the VS donor (24). Specifically, when isolated and fluorescently labeled secreted EVs from primary human VS cells from VS-GH or VS-PH tumors were applied to mouse cochlear explants, neuronal disorganization and neuronal apoptosis was only observed in explants exposed to EVs from VS-PH patients (24). These findings motivated the present series of experiments aimed at identifying the VS-derived EV cargo prompting cochlear damage and determining whether its delivery would cause hearing loss and cochlear damage *in vivo.* To this end, we collected media from cultured primary human VS cells, stratified by the donor patients’ preoperative hearing ability, and isolated EVs from the media to conduct miRNA sequencing of their contents. Following the identification of differentially expressed miRNA in the EVs from VS-GH and VS-PH patients, we designed novel miRNA viral constructs using Anc80L65, an ancestral adeno-associated virus (AAV), for candidate testing in mice to determine their effects on hearing sensitivity and cochlear cell integrity *in vivo*, including following noise exposure.

## 2. Materials and methods

### 2.1. Human VS sample collection

Patients presenting at Massachusetts Eye and Ear (MEE) in Boston, MA for VS resection between April 25, 2014 and October 10, 2014 were included in the study (*N* = 13). Patients’ age at resection, sex, tumor volume, and pre-surgical bilateral audiometric thresholds at six standard frequencies (0.25, 0.5, 1.0, 2.0, 4.0, and 8.0 kHz), and bilateral word recognition (WR) scores were recorded from patients’ medical files. Once the VS was removed, the specimen was transported from the operating room to the laboratory on ice. Primary culture was then performed as we have previously described (24).

### 2.2. Isolation of EVs

The primary human VS cells were cultured for 7 days. The culture medium was then replaced with a culture medium supplemented with 5% EV-depleted fetal bovine serum (FBS; Gibco #16140–071, Thermo Fisher, Waltham, MA, United States), purified by high-speed centrifugation to deplete EVs from the FBS. After 48 h, conditioned media were collected and centrifuged for 10 min at 300 g, then 10 min at 2000 g at 4°C. The supernatant was filtered through a 0.8 μm filter (Millipore; Burlington, MA, United States). EVs were pelleted by centrifugation at 100,000 g for 80 min at 4°C in a Type 70 Ti Rotor (Beckman Coulter, Brea, CA, United States).

The particle distributions (EVs) from the cells were measured using a Nanosight LM20 machine (Malvern Panalytical, Malvern, United Kingdom). A human NF2 VS-derived cell line (HEI-193) immortalized with human papilloma virus E6-7 genes was acquired from House Ear Institute (Los Angeles, CA, United States) (25) and used as a control in the particle distribution measurement (Supplementary Figure S1).

### 2.3. Extraction of RNA from EVs and cells

After centrifugation, the EV pellet was submerged in 4 μL of rDNAase 1 (Life Technologies AM2235; Thermo Fisher) for 1 min and then incubated in 8 μL of Superasin (an Rnase inhibitor; Life Technologies AM2694; Thermo Fisher) for 4 min at room temperature (RT) covered in paraffin. The pellet was resuspended in 700 μL of lysis buffer (QIAzol Lysis Reagent; Qiagen, Germantown, MD, United States) by pipetting. After the Eppendorf tubes were left 3–5 min at RT, 140 μL chloroform was added and mixed by shaking for 15 s, and the phases were allowed to separate for 2–3 min at RT. This process was repeated twice. The tubes were centrifuged for 15 min at 12,000 g at 4°C. The upper aqueous phase was transferred to a new tube and the volume recorded; 1.5x volumes of 100% ethanol were added and mixed thoroughly by pipetting and vortexing. Approximately 500 μL of the sample was immediately transferred to Rneasy Mini spin columns (miRNeasy Kit; Qiagen) and centrifuged with serial applications of washing buffers according to the manufacturer’s instructions. The spin column was transferred to a new 1.5 mL collection tube and rinsed with 32 μL 95°C Rnase-free water for a final elution volume of 30 μL (minus the column volume). The sample was centrifuged for 1 min at 100 g to slowly allow the elution solvent to penetrate the column and then again at 8500 g for 1 min to elute the RNA. This process was repeated with an additional 32 μL 95°C Rnase-free water added to the spin column membrane, in the same collection tube.

For the extraction of RNA from VS cells, after the medium was aspirated and the wells were washed with 1 mL of PBS, 200 μL of 0.25% Trypsin–EDTA (Gibco # 25200–056; Thermo Fisher) was added and allowed to sit for 5 min at 37°C. New media (800 μL/well) was added and mixed by pipetting, then transferred to 1.5 mL sterile Eppendorf tubes. The tubes were centrifuged at 300 g for 2 min at RT, the supernatant was aspirated, and 1 mL PBS was mixed with the pellet. Samples were centrifuged again at 300 g for 5 min at RT and supernatant removed. At this point, the procedures are the same as those described above, beginning with the application of QIAzol Lysis Reagent.

### 2.4. Verification of RNA quality

The quantitative and qualitative analyses of the extracted RNA were performed using the NanoDrop spectrophotometer 2000 (Thermo-Scientific) and Agilent Bioanalyzer 2,100, using the RNA 6000 Pico kit (Agilent; Santa Clara, CA, United States) for RNA from EVs and the RNA 6000 Nano kit (Agilent) for cellular RNA.

### 2.5. sRNAseq by next-generation sequencing and examination of differential expression

All samples were processed using the sRNAseq pipeline developed by the bcbio-nextgen project (26). Raw reads were examined for quality issues using Fast QC to ensure library generation and sequencing were suitable for analysis (27). The 3′ end adapters were trimmed from reads using cutadapt (28), and trimmed reads were aligned to miRbase (29) using SeqBuster (30). miRNA counting was performed with the isomiRs package (31), eliminating any sequence with only 1 count. In a second round of filtering to remove low expressors, a minimum of 2 replicates per condition in every case were required to have ≥3 counts for a given miRNA. Normalization and evaluation of differential expression at the miRNA level were performed with DESeq2 (32).

### 2.6. Construction of viral vectors

Viral vectors were synthesized by the Gene Transfer Vector Core at MEE or the Boston Children’s Hospital Viral Core, as previously published (33). Vectors were sterilized with a 0.22 μm filter and buffered in Dulbecco’s phosphate-buffered saline with 0.001% Pluronic F68 (Gibco #24040032; Thermo Fisher). Aliquots were diluted to an equal concentration (2.84×10^12^ GC/mL) and stored at −80° C.

### 2.7. Posterior semicircular canal injection of viral vectors *in vivo* (mice)

CBA/CaJ (strain #000654) breeding pairs were purchased from Jackson Laboratories (Bar Harbor, ME, United States) and bred at MEE. Neonatal pups were anesthetized by hypothermia and then kept on an ice pack during the procedure. A small incision was made in the left post-auricular region, and the sternocleidomastoid muscle identified. Muscle and soft tissue covering the posterior semicircular canal were gently removed with fine forceps. The semicircular canal wall was punctured with a beveled 35G Hamilton syringe (Hamilton; Reno, NV, United States), the tip was visualized in the canal, and 1.5 μL vector solution was slowly injected over 3 min. The syringe was removed, and the surgical incision closed with 7–0 Ethilon surgical suture (Eticon Inc.; Raritan, NJ, United States). Animals were marked by paw tattoos, allowed to recover on a heating pad, and returned to their litter.

### 2.8. Audiological testing

Cochlear function was evaluated in the mice by measuring auditory brainstem responses (ABRs) and distortion product otoacoustic emissions (DPOAEs) 2–4 days before noise exposure at 6 weeks of age. Two weeks after noise exposure, cochlear function was re-assessed by ABR and DPOAE, the animals were sacrificed, and their cochleae were collected for histological analysis. ABR and DPOAE were recorded as previously described (34); mice were anesthetized with ketamine (100 mg/kg) and xylazine (10 mg/kg) administered intraperitoneally during testing. Cochlear function testing and data quantification were performed by the researcher blinded to the treatment group.

ABR responses to 5 ms tone pips were measured between subdermal electrodes (positive behind the ipsilateral pinna, negative at the vertex, and ground at the tail), amplified 10,000 times, and filtered (0.3–3.0 kHz). For each frequency and sound level, 512 responses were recorded and averaged using custom LabVIEW data-acquisition software run on a PXI chassis (National Instruments Corp., Austin, TX, United States). The ABR waveforms were stacked from lowest to highest SPL and visually inspected to define threshold as the first level at which a repeatable wave I was detected. ABR data were acquired in 5-dB intensity increments below threshold and at 5- to 10-dB increments above threshold.

For DPOAE measurement, a custom acoustic system was used consisting of two miniature earphones serving as sound sources (CDMG150 008-03A, CUI) and a microphone (FG-23329-PO7; Knowles, Itasca, IL, United States) coupled to a probe tube to measure sound pressure near the eardrum. DPOAEs were measured as ear canal pressure in response to two tones presented into the ear canal (f1 and f2, with f2/f1 = 1.2) at half octave steps, from f2 = 5.66–45.25 kHz, and in 5-dB intensity increments from 15 to 80 dB SPL.

### 2.9. Noise exposure

Mice were exposed to octave-band noise (8–16 kHz) for 2 h at 100 dB SPL in a reverberant, acoustically transparent wire box on a rotating platform. Animals were awake and unrestrained during noise exposure. The noise was created digitally using a fifth-order Butterworth filter, amplified through a power amplifier (Crown D75A; Crown Audio, Los Angeles, CA, United States), and delivered by a loudspeaker (JBL2446H; JBL, Los Angeles, CA, United States) coupled to an exponential horn in the roof of the box. Exposure levels were measured in each cage with a 0.25-inch Brüel and Kjaer (Nærum, Denmark) condenser microphone.

### 2.10. Preparation of cochlear whole mounts

Two weeks after noise exposure, the mice were deeply anesthetized, intracardially perfused with 4% paraformaldehyde (PFA, #P6148; Sigma-Aldrich, St. Louis, MO, United States), and both cochleae were extracted. The round and oval window membranes were punctured and gently perfused with PFA. Cochleae were post-fixed for 2 h in 4% PFA and decalcified in 0.12 M EDTA (#17892, Thermo Fisher) for 48–72 h. The cochleae were microdissected into 4 to 6 separate pieces to prepare whole mounts of the organ of Corti. Pieces were incubated in 30% sucrose for 15 min at room temperature and then frozen at −80° C to permeabilize the tissue. The pieces were blocked with 5% normal horse serum (NHS; #16050130, Thermo Fisher) and 1% Triton X-100 (#NC9903183; Integra Chemical, Kent, WA, United States) in PBS (#10010023, Thermo Fisher) for 30 min at RT.

### 2.11. Immunohistochemistry and quantitative confocal immunofluorescence microscopy

Cochlear samples were immunostained overnight at RT with rabbit anti-myosin 7A (1:200, #25–6,790, Proteus Biosciences, Ramona, CA, United States) to label hair cells and mouse (IgG1) anti-CtBP2 (C-terminal binding protein) at 1:500 (#612044, BD Transduction Labs, CA, United States) to label pre-synaptic ribbons, diluted in 1% NHS (#16050130, Thermo Fisher) and 0.3% Triton X-100. After washing in PBS three times, cochlear pieces were incubated in Alexa Fluor 647-conjugated goat anti-rabbit antibody at 1:1000 (#A-21245, Thermo Fisher) and Alexa Fluor 568-conjugated goat anti-mouse (IgG1) at 1:1000 (#21124, Thermo Fisher) twice for 1 hour. Nuclei were stained with Hoechst 33342 at 1:10000 (#62249, Thermo Fisher) and the specimen washed three times in PBS and mounted with VECTASHIELD mounting medium (Vector Labs, Newark, CA, United States). A cochlear frequency map was created by imaging specimens at low magnification (10X objective) and then applying a custom ImageJ plug-in developed at MEE.^1^ Cochlear whole mounts were subsequently imaged with a confocal microscope (Zeiss, Jena, Germany; LSM 880 confocal microscope) and a glycerol-immersion 63X objective and 2X digital zoom at log-spaced cochlear frequency regions corresponding to 8, 16, and 32 kHz.

### 2.12. Ethics statements

Clinical data and samples were collected and used according to a protocol approved by the institutional review boards of MEE (#196424, approved December 10, 2013) and all procedures were in accordance with the Helsinki Declaration of 1975. All patients provided informed consent prior to study inclusion. Patients did not receive compensation for their participation in the study. The experimental procedures in mice were approved by the Institutional Animal Care and Use Committee of MEE (ACC# 15–003) and conducted in accordance with the NIH Guide for the Care and Use of Laboratory Animals.

## 3. Results

### 3.1. VS specimens stratified by donors’ pre-surgical hearing

**Figure 1:**
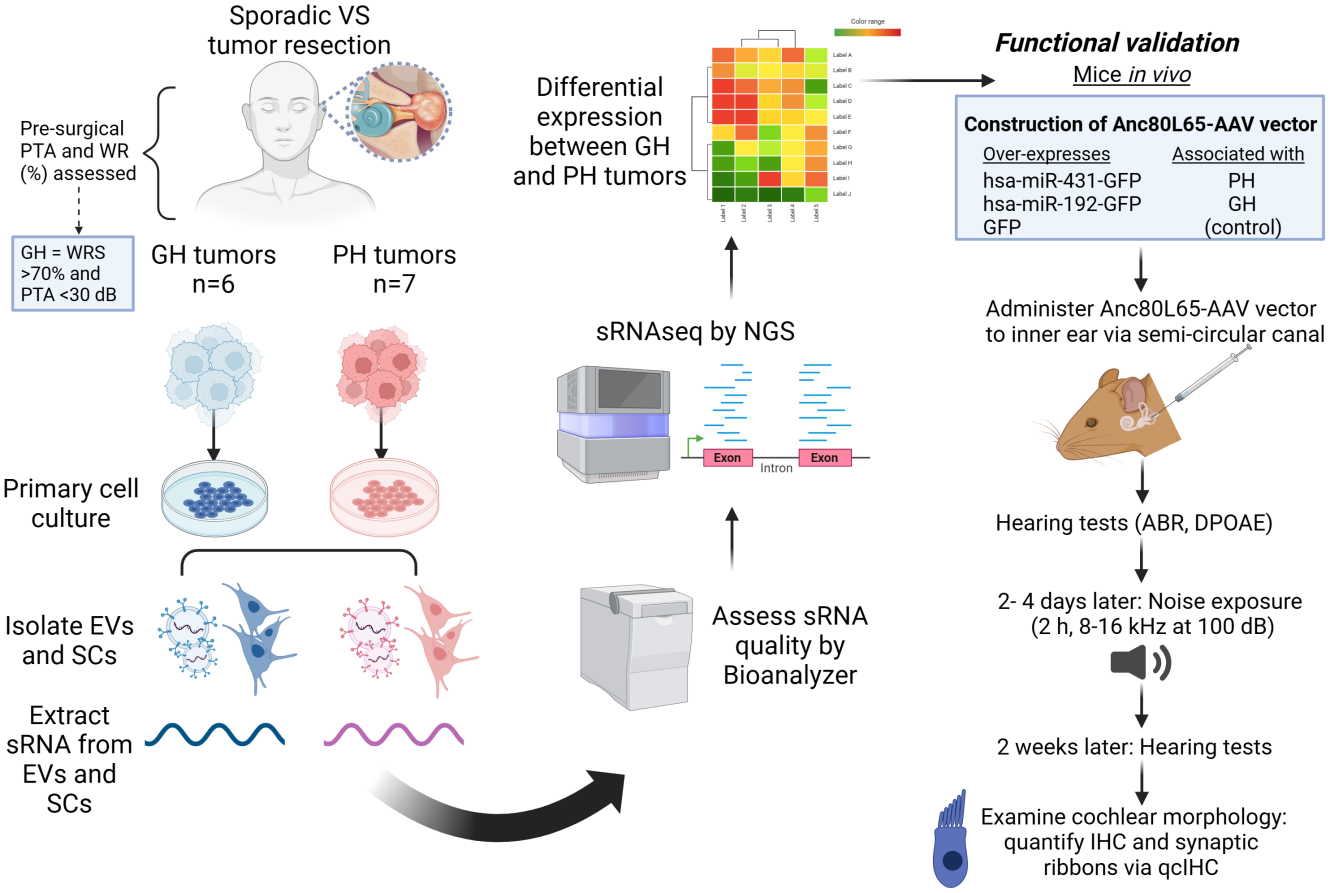
Experimental overview. ABR, auditory brainstem response; dB, decibels; DPOAE, distortion product otoacoustic emissions; EV, extracellular vesicles; GFP, green fluorescent protein; GH, good hearing; h, hour; IHC, inner hair cell; kHz, kilohertz; miRNA, micro RNA; PH, poor hearing; PTA, pure tone average; RNAseq, RNA sequencing; qcIHC, quantitative confocal immunohistochemistry; SC, schwannoma cells; sRNA, short RNA; VS, vestibular schwannoma; WRS, word recognition score. Figure made using Biorender (www.biorender.com).

VS tumor samples from 13 people with sporadic VS were collected during indicated tumor resection surgeries (Figure 1). Patients’ age at resection, sex, tumor volume and dimensions, and pre-surgical bilateral audiometric thresholds [pure tone average (PTA)] and WR scores were recorded (Table 1). Patients were categorized as having PH or GH based on their pre-surgical PTA threshold and WR score, where PH was defined as PTA >30 dB and a WR score of <70% according to the American Academy of Otolaryngology’s classification system (35). Among the 13 included patients, 6 were categorized as VS-GH and 7 were categorized as VS-PH.

**Table 1:** Characteristics of the VS tumor tissue donors and quantification of RNA extracted from VS tumor cells and EVs.

					Tumor characteristics	Hearing tests		Bioanalyzer		
	Hearing category^a^	Sex	Age (y)	Location (side)	Size, all 3 linear dimensions (mm)^b^	PTA (dB)	WR (%)	Cells RIN^c^	[RNA], μg/μl	
Sample #									Cells	EVs
Used in the analyses of VS cell and EV RNA										
1	GH	F	46	Right	18 TV × 17 AP × **24** SI	12	94	9.9	761.00	1.89
2	GH	F	34	Right	**22** TV × 19 AP × 20.5 SI	5	100	8.7	124.00	4.44
3	GH	F	43	Left	**14** TV × 8 AP × 8 SI	10	100	10	296.00	2.96
4	GH	F	55	Right	**22** TV × 20 AP × 21 SI	12	98	9.1	171.00	4.30
5	GH	F	51	Left	**27** TV × 21 AP × 22 SI	12	92	8.8	82.00	15.35
6	PH	M	58	Right	25 TV × 31.8 AP × **34** SI	50	48	8.4	92.00	2.06
7	PH	F	54	Right	**29** TV × 22 AP × **29** SI	35	94	9.9	206.00	10.80
8	PH	F	45	Right	**13** TV × 5 AP × 5 SI	95	0	10	344.00	3.80
9	PH	F	45	Left	**24** TV × 12 AP × 10 SI	58	0	8.1	52.00	2.00
Used in the analysis of EV RNA only										
10	PH	F	55	Right	**25** TV × 13 AP × 12 SI	40	20	–	–	7.10
11	PH	M	56	Left	**27** TV × 15 AP × 13 SI	37	58	–	–	5.00
12	PH	M	43	Left	27 TV × **36** AP × 26.6 SI	50	92	–	–	8.60
13	GH	F	22	Right	**30** TV × 28 AP × 29 SI	12	96	–	–	12.07

Sufficient quality and concentration of RNA were derived from both VS cells and EVs for 9 VS tissue samples and from only EVs for 4 samples. AP, anterior–posterior; dB, decibel; EVs, extracellular vesicles; F, female; GH, good hearing; M, male; PH, poor hearing; PTA, pure tone average; RIN, RNA integrity number; SI, superior–inferior; TV, transverse; VS, vestibular schwannoma; WR, word recognition; y, years.

a PH was defined as WR score < 70% or PTA > 30 dB; otherwise, patients were categorized as GH.

b The longest linear dimensions are in bold.

c The bioanalyzer provides a RIN, an objective metric of total RNA quality ranging from 10 (highly intact) to 1 (completely degraded). Samples with a “–” had a RIN below the quality threshold of 8.0 and were not included in the analysis of cell RNA.

### 3.2. Isolation of EVs from VS cell-conditioned media

**Figure 2:**
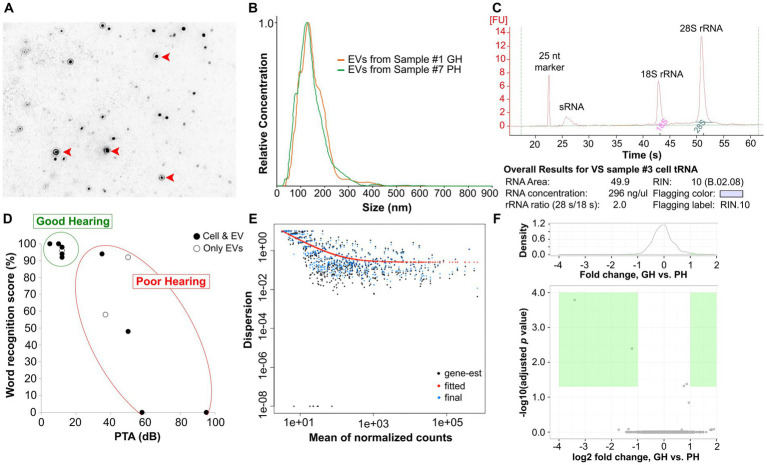
Isolation and purification of EVs from conditioned media and VS cells. EVs (red arrowheads) were isolated from VS cell-conditioned media via ultracentrifugation, verified by NanoSight **(A)**. The relative concentrations and sizes of EVs were similar across samples **(B)**. The quality of total RNA extracted from EVs was assessed via a Bioanalyzer. An example (VS sample #3) of the Bioanalyzer results is presented in **(C)** and the results for all samples are listed in Table 1. The distribution of the preoperative hearing test scores of VS-PH and VS-GH patients from which cell and/or EV miRNA were derived is shown in **(D)**. EV sRNA was derived from all samples while VS cell-derived sRNA was obtained from 4 VS-PH samples and 5 VS-GH samples. PH was defined as word recognition score < 70% and PTA >30 dB; otherwise, patients were categorized as GH. sRNA with RNA integrity number (RIN) ≥8.0 was sequenced using the Illumina HiSeq2500 platform and the miRNA dispersion plotted by the mean of the normalized counts **(E)**. miRNA expression levels were compared between VS-GH and VS-PH groups to identify differential expression of individual miRNAs **(F)**. dB, decibel; EV, extracellular vesicle; gene-est, gene expressed sequence tag; GH, good hearing; miRNA, micro RNA; nt, nucleotides; PH, poor hearing; PTA, pure tone average; rRNA, ribosomal RNA; sRNA, small RNA; VS, vestibular schwannoma.

The VS-GH and VS-PH specimens were separately cultured in EV-depleted culture media for 48 h to generate conditioned media as previously described (24). Primary VS cell cultures are representative of their parent tumors, and this method provided the ability to study purified tumor cells and their EVs (24). EVs were isolated from VS cell-conditioned media using ultracentrifugation, which was verified by transmission electron microscopy (Figure 2A). The relative concentrations and size distributions of EVs were similar across primary VS cell cultures and a NF2 VS-derived immortalized cell line (HEI-193) used as a control, with most cell-derived EVs measuring ~100–200 nm (Figure 2B; Supplementary Figure S1).

### 3.3. Extraction of small RNA (sRNA) from EVs

Total RNA (including sRNA) was extracted from EVs isolated from VS cell-conditioned media and parent cells and the quality assessed with a Bioanalyzer (see example in Figure 2C). Exosomal sRNA was identified as measuring 200–300 nucleotides (nt) with the Bioanalyzer, as described in the Methods. The Bioanalyzer provides an RNA integrity number (RIN), an objective metric of total RNA quality ranging from 10 (highly intact) to 1 (completely degraded). The concentrations and RINs of the individual RNA samples are listed in Table 1; samples below the quality threshold of RIN ≥ 8.0 were excluded from the analyses. EV sRNA was isolated from all samples while VS cell-derived sRNA was obtained from 4 VS-PH samples and 5 VS-GH samples (Figure 2D; Table 1).

### 3.4. Next-generation sequencing of sRNA and candidate miRNA

**Figure 3:**
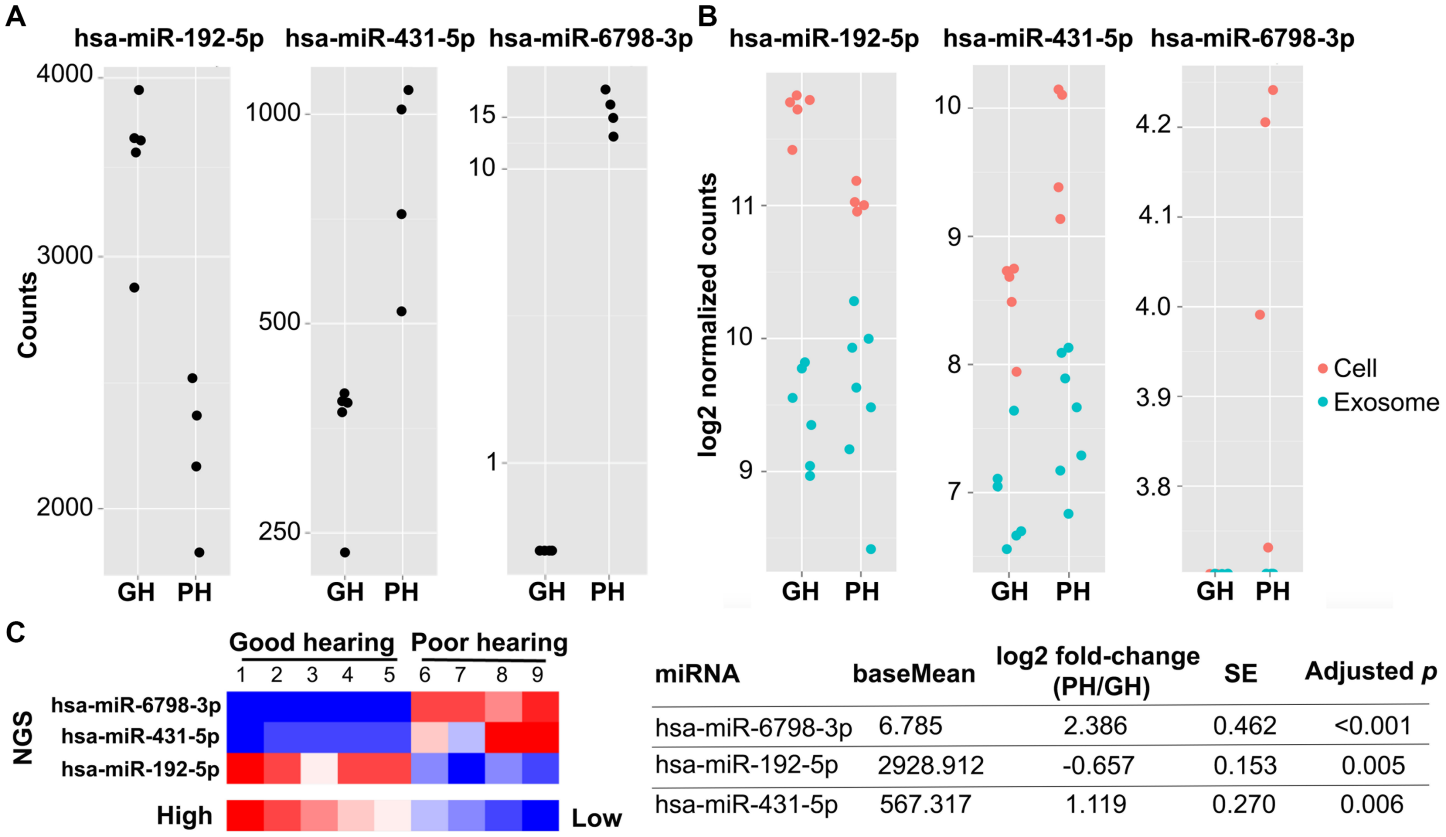
Next-generation sequencing of miRNA from individual VSs from patients with good (*n* = 5) and poor (*n* = 4) preoperative hearing. The expression levels of hsa-miR-6798-3p, hsa-miR-431-5p, and hsa-miR-192-5p significantly differed between VS-GH and VS-PH cells (table inset). The total and log2 normalized counts of these miRNA are plotted by hearing category in **(A)** and **(B)**, respectively. In **(B)**, red dots represent samples from VS cells and blue dots represent samples from EVs (exosomes). The heat map **(C)** displays enrichment of the miRNA in the groups, with higher levels of hsa-miR-6798-3p and hsa-miR-431-5p in VS-PH samples, and higher levels of hsa-miR-192-5p in VS-GH samples. There were no differentially expressed miRNA in EVs from VS-GH vs. VS-PH samples. EV, extracellular vesicle; GH, good hearing; miRNA, micro RNA; NGS, next-generation sequencing; PH, poor hearing; SE, standard error.

sRNA meeting the RIN quality threshold was sequenced using next-generation sequencing (NGS) (Illumina HiSeq2500 platform), and the miRNA dispersion plotted by the mean of the normalized counts and analyzed as described in the Methods (Figure 2E). A total of 395 miRNAs were detected in EVs and 564 miRNAs were detected in VS cells (Supplementary Tables S1, S2). To identify EV-delivered cargo that may be mediating VS-induced SNHL, we sequenced miRNA from EVs and VS cells classified according to the donor patients’ levels of hearing loss (GH or PH). The expression levels of EV- and VS cell-derived miRNA were then compared between VS-GH and VS-PH samples to categorize miRNA as being prevalent in VS-GH or VS-PH and identify significant differences across samples (Figure 2F). We identified three miRNAs with significantly different expression in VS-PH vs. VS-GH tumor cells: hsa-miR-6798-3p (log^2^ fold change [FC]: 2.39), hsa-miR-192-5p (log^2^ FC: −0.66), and hsa-miR-431-5p (log^2^ FC: 1.12; all adjusted *p* < 0.01) (Figures 3A,B and table inset). hsa-miR-6798-3p and hsa-miR-431-5p were enriched in VS-PH cells while hsa-miR-192-5p was enriched in VS-GH cells (Figure 3C). No miRNAs were differentially expressed in EVs.

### 3.5. Characterization of miRNA candidates *in vivo*

**Figure 4:**
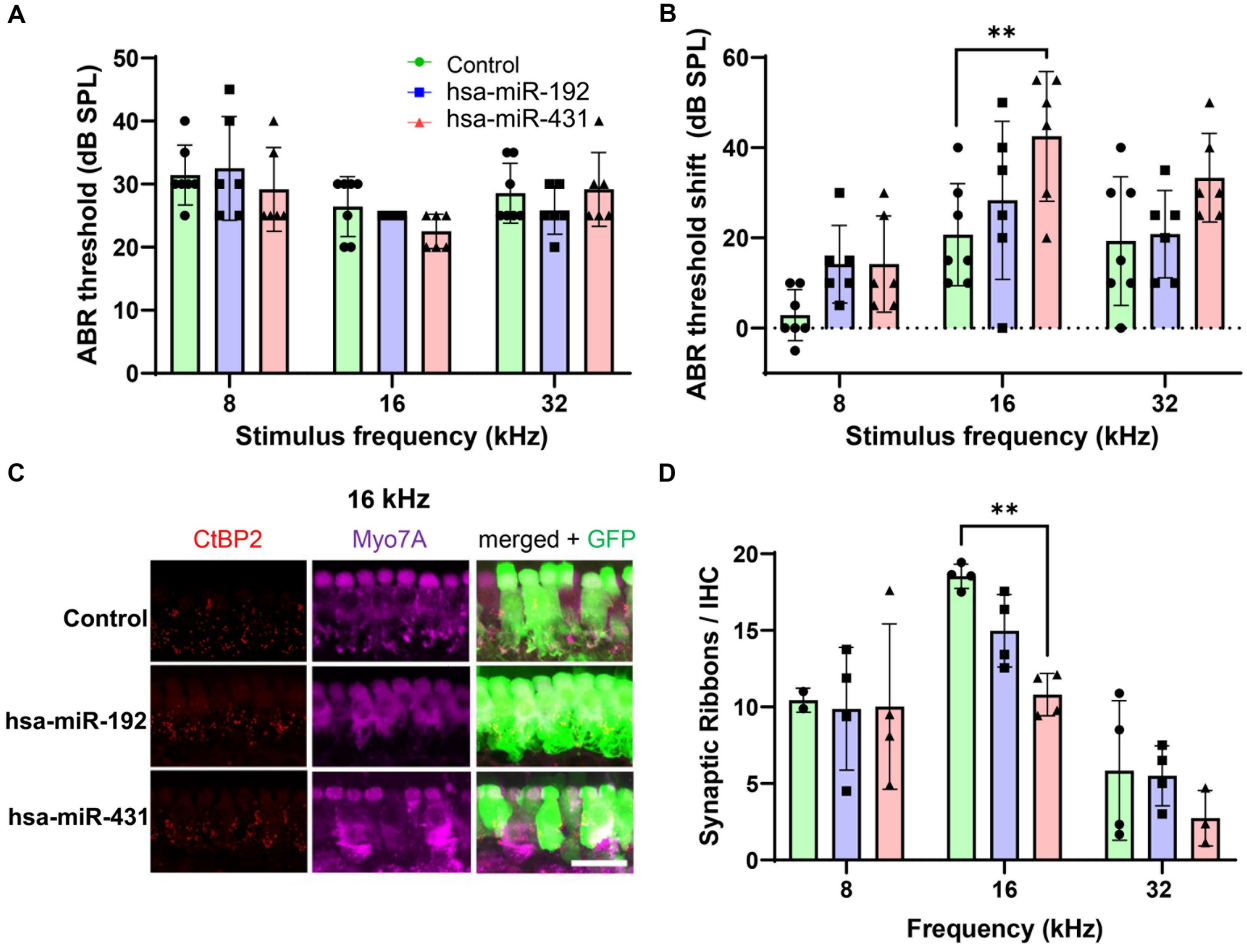
Mice overexpressing hsa-miR-431, associated with poor hearing in human VS, exhibit greater loss of hearing and cochlear synapses after noise exposure. Inner ears of postnatal day 1–2 wildtype mice were transduced with Anc80L65 AAV overexpressing hsa-miR-431-GFP, hsa-miR-192-GFP, or GFP only (control). Approximately 6 weeks later, ABR **(A)** measurements 2–4 days before noise exposure (100 dB at 8–16 kHz for 2 h) confirmed there were no significant differences in hearing between the groups. Two weeks after noise exposure, hsa-miR-431 overexpressing mice showed a 20 dB ABR threshold shift at 16 kHz **(B)** that was significantly higher compared to that of the control group (*p* < 0.01). Using quantitative confocal immunohistochemistry, we observed that the numbers of synaptic ribbons and IHCs were significantly lower among hsa-miR-431 overexpressing mice compared to the GFP-only control group in the region corresponding to 16 kHz **(C,D)** following noise exposure. Hair cell transduction was observed to be similar across the three vectors. In **(A,B,D)**, green (circles) indicate control group, blue (squares) indicates hsa-miR-192, and red (triangles) indicates hsa-miR-431. AAV, adeno-associated virus; ABR, auditory brainstem response; CtBP2, C-terminal binding protein 2; db, decibel; DPOAE, distortion product otoacoustic emissions; GFP, green fluorescent protein; GH, good hearing; IHC, inner hair cell; kHz, kilohertz; Myo7a, myosin 7a; PH, poor hearing; SE, standard error; SPL, sound pressure level; VS, vestibular schwannoma. *p < 0.05, **p < 0.01. Scale bar, 20 μm **(C)**.

To test the effect of the candidate miRNA on the mammalian inner ear *in vivo*, two AAV vectors associated with PH and GH, respectively, were constructed: hsa-miR-431-overexpressing AAV2/Anc80L65.AAP.U6.mir413.CMV.EGFP.SVPA and hsa-miR-192-overexpressing AAV2/Anc80L65.AAP.U6.mir192.CMV.EGFP.SVPA. An additional vector (AAV2/Anc80L65.CAG.GFP.WRPE) was constructed as a GFP-only control. Postnatal day (P) 1–2 CBA/CaJ mouse pups from four litters were randomly assigned to an AAV injection group: GFP-only control (*n* = 7), hsa-miR-192 (*n* = 6), or hsa-miR-431 (*n* = 6). The mice were injected intracochlearly via the semicircular canal with 1.5 μL vector solution over 3 min. At ~6 weeks of age (post-injection), baseline hearing was evaluated in the mice by measuring ABRs and DPOAEs, which reflect auditory evoked potentials and OHC function, respectively. Hearing was normal and there were no significant differences in hearing between the groups (Figure 4A; Supplementary Figure S2A).

VS-mediated SNHL may be prompted by ototoxic stress such as from noise or other insults to the inner ear. Thus, to determine whether candidate miRNA expression in the cochlea sensitizes the ear to noise trauma, all mice underwent noise exposure (100 dB SPL at 8–16 kHz for 2 h) 2 to 4 days after assessment of their hearing function. At 2 weeks post-noise exposure, hearing was again assessed with ABR and DPOAE. Mice injected with the AAV overexpressing miR-431 had a 20 dB ABR threshold shift from baseline at 16 kHz that was significantly higher compared to the GFP-only control group (*p* < 0.01) (Figure 4B). Mice injected with the AAV overexpressing miR-192 did not have a significant ABR threshold shift from baseline in comparison with the GFP-only control group. There were no significant differences in the post-noise exposure DPOAE threshold shifts between the GFP-only control group and either of the experimental groups (Supplementary Figure S2B).

The mice were then sacrificed, and the extracted cochleae underwent immunohistochemistry to enable counting of IHC (myosin 7A-positive) and pre-synaptic ribbons (CtBP2-positive) (16 kHz region displayed in Figure 4C). Following noise exposure, the number of synaptic ribbons per IHC were significantly lower among miR-431 overexpressing mice compared to the GFP-only control group in the region corresponding to 16 kHz (*p* < 0.01) (Figure 4D). There were no significant differences in the number of synaptic ribbons per IHC between the miR-192 overexpressing mice compared to the GFP-only control group.

## 4. Discussion

In this study, miR-431-5p was found to be significantly more abundant in VS tumor cells from patients with poor hearing than those with good hearing, pointing to a potential miRNA candidate contributing to VS-associated hearing loss. Although viral overexpression of miR-431 in the inner ear of mice produced no initial negative effects on hearing, following noise exposure, these mice exhibited significantly greater hearing loss, as well as greater loss of IHC synapses in the cochlear region most affected by the noise trauma. These results suggest that miR-431, enriched in the VS tumors of patients with poor hearing, may sensitize the cochlea to stress-induced trauma, including from noise, and the development of subsequent hearing loss. Our findings provide mechanistic insight into the clinical observation that patients with VS demonstrate increased susceptibility to acoustic trauma (36), and that mid-frequency trough-shaped audiograms, which are characteristic of acoustic trauma, are common in VS patients (37). It is also interesting to note that case–control and population-level studies have observed increased risk of VS with loud noise exposure (38–40), thus the etiological link between noise trauma, the VS tumor, and VS-mediated SNHL merits further study.

While there are two prior reports identifying a role for miR-431 in hearing function and dysfunction (41, 42), there are no reports on its role in VS-induced hearing loss or acoustic trauma. miR-431 has been localized in both the mammalian cochlea and vestibular organs (41), and is highly expressed in the SGNs of newborn mice, with its expression decreasing into adulthood (42). Although transgenic mice overexpressing miR-431 appear to have structurally normal cochleae, they have significantly higher (worse) ABR thresholds than wildtype mice due to lower density and less branching of SGNs (42). Moreover, cochlear cultures from miR-431-overexpressing mice displayed fewer mature SGNs and shorter axons than controls (42). In contrast to the transgenic mice in Fan et al. which overexpressed miR-431 during cochlear development, the mice in our study were injected with a vector to overexpress miR-431 at 6 weeks of age, when SGNs and their synaptic connections with IHC would be mature. Thus, we hypothesize that synaptic function is impaired in our miR-431-injected mice, but the phenotype of worse ABRs only emerges post-noise trauma that stresses synapses between the SGNs and IHCs. This would explain why our miR-431-overexpressing mice did not demonstrate an ABR shift shortly after injection, while the transgenic mice in Fan et al. demonstrated an ABR shift at 13 weeks post-natal. Notably, a microarray study comparing miRNA levels between VS and control cranial nerve tissue reported that miR-431 was the top differentially upregulated miRNA in VS, although no association with hearing was investigated (43). In that study, around 40 miRNA located in the chromosomal 14q32 region were upregulated in VS (miR-431 is located at 14q32.2 specifically) (43). Deletions or alterations of this area have been implicated in rare cases of syndromic hearing loss in humans (44, 45), and in the formation of tumors like gliomas (46).

An mRNA target of miR-431, as suggested by bioinformatic and molecular assays, is *Eya4*, with higher expression of miR-431 resulting in decreased production of the protein EYA4 (41, 42). *Eya4*^−/−^ mice exhibit profound hearing loss (47) and *EYA4* mutations in humans are linked to autosomal-dominant non-syndromic hearing loss, among other defects (48–50). Interestingly, *EYA4* appears to remain important in adulthood for the maintenance of cochlear function in mammals. In rats, *Eya4* expression persists in the organ of Corti and spiral prominence into the postnatal period (50), and people with certain *EYA4* mutations exhibit late-onset hearing loss (50, 51). Further, *EYA4* mutations have been associated with increased vulnerability to noise-induced hearing loss in occupational settings (52, 53). Thus, in addition to its role in cochlear development and maturation, *EYA4* may have a role in the maintenance of mature SGN survival following a cochlear insult. In this study, the hearing deficits and loss of ribbon synapses in the miR-431-overexpressing mice following noise exposure may be partially explained by the depletion or suppression of *EYA4*.

miR-431 also interacts with *Smad4* RNA, which is required for canonical signaling by TGF-β, a potent cytokine and growth factor (54, 55). Specifically, elevation of miR-431 decreases SMAD4 levels and thereby reduces inhibition of TGF-β, while decreasing miR-431 has the opposite effect (54, 56–58). The TGF-β/SMAD4 signaling pathway controls signal transduction from the cell membrane to the nucleus and is involved in many cellular processes such as cell proliferation, migration/motility, differentiation, apoptosis, inflammatory/immune response, and tumor formation and progression (55). Both type I and II receptors of TGFβ-1 (regulated by SMAD4) have been identified in the modiolus, organ of Corti, and lateral wall of the mammalian cochlea (59). TGF-β isoforms are expressed in the developing mammalian cochlea in distinct patterns and are involved in SGN and ribbon synapse formation and survival (60, 61). Similarly, conditional knockouts of *Smad4* display disruption of ribbon synapses and auditory neuropathy (62, 63). In humans, the rare autosomal disorder Myhre syndrome is caused by a gain-of-function mutation in *SMAD4,* which is associated with hearing loss among other musculoskeletal anomalies (64, 65), as well as reports of unilateral VS (66). In animal models, TGF-β1 rapidly increases in the cochlea following noise exposure, suggesting that it participates in the immunological response to noise trauma, before declining over the next 7 days (61). However, sustained high levels of TGF-β1 were associated with greater noise-induced cochlear trauma, which could be reversed with inhibitors (61). Interestingly, intracochlear injection of Ad.*GDNF* plus Ad.*TGFβ-1* in guinea pigs resulted in greater preserved hearing, IHCs, and OHCs after ototoxic drug administration compared with animals injected with Ad.*GDNF* alone, but this was accompanied by proliferative fibrosis (59). Thus, the role of TGFβ-1/SMAD4 signaling in the cochlea may depend on the presence of other molecules like GDNF or miR-431.

Additionally, increased miR-431 could prompt ototoxicity via altering the role of TGF-β/SMAD4 in acid extrusion from the VS tumor. Dysregulated pH homeostasis due to elevated metabolic acid production and extrusion is a notable feature of the microenvironment of solid tumors (67–69), and can lead to an acidic extracellular pH in the tumor while cytoplasmic intracellular pH remains normal or even slightly alkaline (67, 68). Ion transporters involved in acid extrusion are regulated by TGF-β in a SMAD4-dependent fashion and have been implicated in other solid tumors (70). Furthermore, Merlin—the gene product disrupted in VS—has also been shown to regulate ion transporters in cancer (70, 71), which may further increase the acidification of the tumor and negative impacts to the cochlear microenvironment. Considering these findings along with our own data, an overabundance of miR-431 may enhance noise-induced cochlear injury in myriad ways, including miR-431 depletion of EYA4 as well as dysregulation of the TGF-β/SMAD4 signaling pathway. These two mechanisms are involved in the maintenance of mature SGNs, ribbon synapses, and other cochlear cells, and may be a novel mechanism for VS-associated hearing loss.

A limitation of our study is that it focused on acoustic trauma as a sensitizer of the inner ear to postnatal miR-431 overexpression. Future experiments should define whether aging or insults other than noise, such as exposure to ototoxic drugs or tumor-secreted factors, also sensitize the cochlea to damage during miR-431 overexpression. Additionally, future prospective studies examining the levels of miR-431 in the blood, cerebrospinal fluid, and inner ear fluids (i.e., perilymph) of patients with VS and/or hearing loss are recommended to understand how it may circulate within the body to potentially impact hearing. Moreover, future large-scale epidemiologic studies should determine whether noise trauma is a modifiable risk factor for VS-induced hearing loss. The results of these studies, combined with the current results, may motivate future development of molecular therapeutics that inhibit miR-431 in order to prevent VS-induced hearing loss.

## Data availability statement

The data presented in this study are deposited in the Stankovic Lab website, accessible directly at: https://stanfordmedicine.app.box.com/s/sq3nidp5l141qu27le3qvmx909wywsex.

## Ethics statement

The studies involving humans were approved by the institutional review boards of Massachusetts Eye and Ear (#196424). The studies were conducted in accordance with the local legislation and institutional requirements. The participants provided their written informed consent to participate in this study. The animal study was approved by Institutional Animal Care and Use Committee of Massachusetts Eye and Ear (ACC# 15-003). The study was conducted in accordance with the local legislation and institutional requirements.

## Author contributions

TF: Formal analysis, Methodology, Writing – original draft, Writing – review & editing, Data curation, Investigation, Validation, Visualization. RS: Formal Analysis, Investigation, Methodology, Visualization, Writing – original draft, Writing – review & editing. S-YK: Methodology, Writing – review & editing, Validation. VS: Methodology, Writing – review & editing, Investigation. LP: Investigation, Methodology, Writing – review & editing, Formal analysis. RK: Formal analysis, Investigation, Methodology, Writing – review & editing. LL: Investigation, Methodology, Writing – review & editing. SB: Writing – review & editing, Visualization, Writing – original draft. KS: Writing – original draft, Writing – review & editing, Conceptualization, Formal analysis, Funding acquisition, Methodology, Project administration, Resources, Software, Supervision.

## Footnotes

^1^https://www.masseyeandear.org/research/otolaryngology/eaton-peabody-laboratories/histology-core
