# Supplementary material for: miR-431 secreted by human vestibular schwannomas increases the mammalian inner ear’s vulnerability to noise trauma
Source: Front Neurol. 2023 Oct 9;14:1268359. doi: 10.3389/fneur.2023.1268359 (PMC10598552; doi:10.3389/fneur.2023.1268359)
Supplement: Supplementary file 3 [file Data_Sheet_1.docx]

Supplementary Material

# Supplementary Figures

**Supplemental Figure 1. Particle analysis of exosomes from control HEI-193 cells**

**
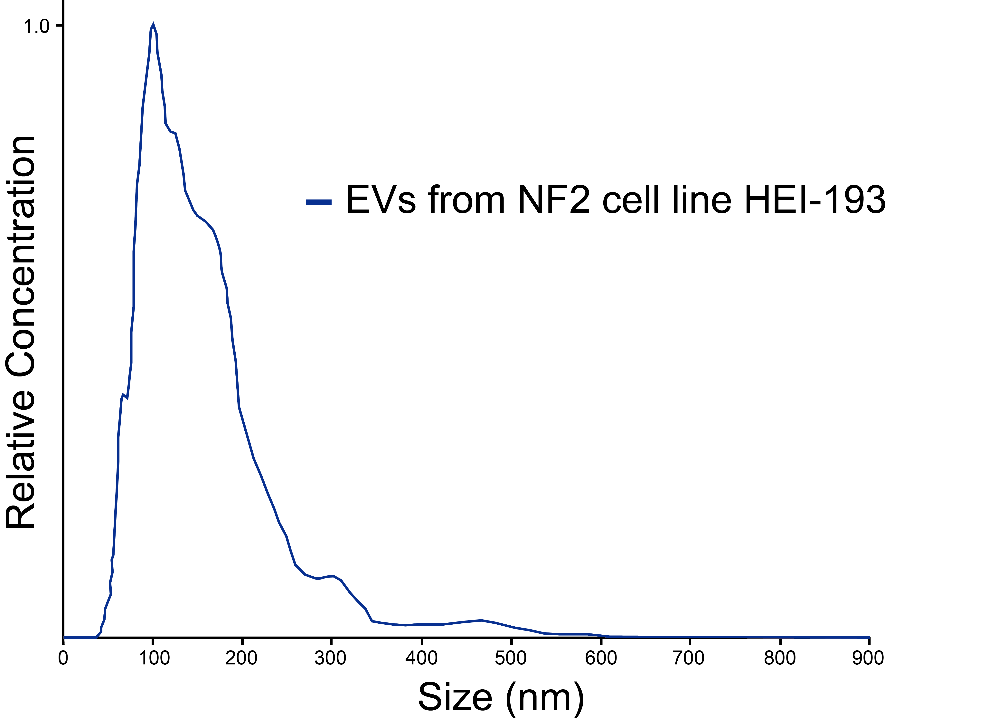
**

EVs from a human NF2 VS-derived cell line (HEI-193) were used as a control to determine the relative concentrations and sizes of EVs in the experimental samples (see **Figure 2b**). Abbreviation: EVs, extracellular vesicles; NF2, neurofibromatosis 2.

**Supplemental Figure 2. DPOAE measurements, before and after noise exposure, of mice that received AAV overexpressing candidate miRNA**

**
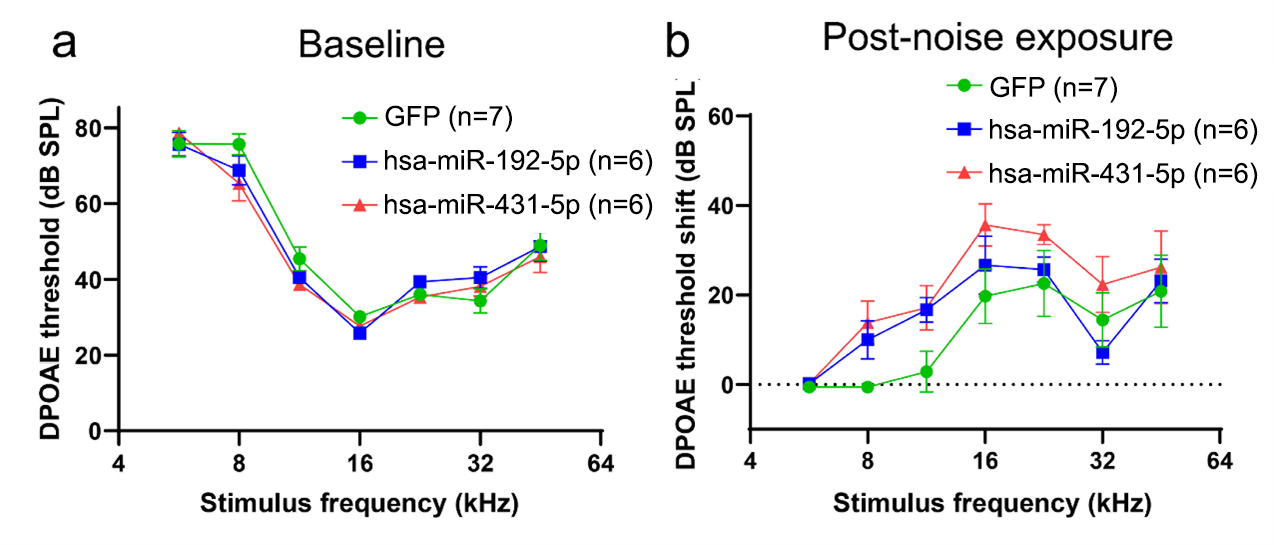
**

Inner ears of postnatal day 1-2 wildtype mice were transduced with Anc80L65 AAV overexpressing hsa-miR-431-GFP, hsa-miR-192-GFP, or GFP only (control). DPOAE measurements were acquired 2-4 days before (**a**) and two weeks after (**b**) noise exposure (100 dB at 8-16 kHz for 2 hours). There were no significant differences in the DPOAE measurements between groups at two weeks post-noise exposure. Abbreviations: AAV, adeno-associated virus; db, decibel; DPOAE, distortion product otoacoustic emissions; GFP, green fluorescent protein; kHz, kilohertz; miRNA, microRNA; SPL, sound pressure level.
